# Low occupational physical activity is associated with incident type 2 diabetes in overweight and obese individuals: a population-based cohort study

**DOI:** 10.1186/s12889-025-22534-5

**Published:** 2025-04-14

**Authors:** Maria Brännholm Syrjälä, Melony Fortuin-de Smidt, Frida Bergman, Maria Nordendahl, Julia Otten, Rebecka Renklint, Olov Rolandsson, Viktoria Wahlström, Patrik Wennberg

**Affiliations:** Department of Public Health and Clinical Medicine, Umeå University, Umeå, Sweden

**Keywords:** Prevention, Type 2 diabetes, Occupational physical activity, Occupational sitting, Obesity, Overweight, Public health

## Abstract

**Background:**

Leisure-time physical activity decreases the risk of type 2 diabetes. Whether occupational physical activity affects the risk of type 2 diabetes is still not fully understood.

The primary aim of this study was to investigate the association between occupational physical activity and 10-year diabetes incidence in a general adult population in Northern Sweden. The secondary aim was to explore the moderating role of BMI on this association.

**Methods:**

This population-based, longitudinal cohort study included 16,282 diabetes-free individuals aged 28–52 years who participated in a cardiovascular intervention programme in Northern Sweden, and who reported the same occupational physical activity level at baseline and at 10-year follow-up. Incident type 2 diabetes was diagnosed based on oral glucose tolerance testing or a register-based diagnosis. Occupational physical activity was self-reported and categorized as: a) Low: ‘Sedentary or standing’ or ‘Light but partly physically active’, b) Moderate: ‘Light and physically active’, or c) High: Sometimes physically strenuous or ‘Physically strenuous most of the time’. Odds ratios (OR) and 95% confidence intervals (CI) for incident diabetes were calculated using multivariable logistic regression analysis, adjusting for age, sex, smoking, education level, family history of diabetes, country of birth, intake of fruits and vegetables, leisure-time physical activity, prediabetes and BMI. Potential interactions between BMI category and T2D were tested using interaction terms in the multivariable model.

**Results:**

Six hundred twenty-four individuals developed type 2 diabetes in the 10 years between the first visit and the follow-up. A significant moderation effect of BMI on occupational physical activity was found (*p* = 0.01). Having a low level of occupational physical activity, compared with a moderate level of occupational physical activity, was associated with an increased risk of incident type 2 diabetes in overweight and obese individuals (OR 1.46, 95% CI 1.09–1.96), but not in those with normal weight (OR 0.80, 95% CI 0.52–1.23). High level of occupational physical activity was not associated with type 2 diabetes (OR 1.12, 95% CI 0.82–1.54).

**Conclusions:**

Low occupational physical activity was associated with incident type 2 diabetes in overweight and obese individuals. Public-health efforts may benefit from encouraging less sitting and standing and more light physical activity during the workday.

**Supplementary Information:**

The online version contains supplementary material available at 10.1186/s12889-025-22534-5.

## Background

Type 2 diabetes (T2D) is a major contributor to the global disease burden that, according to the Global Burden of Disease Study 2019, affects adults worldwide [1]. Despite improvements in life expectancy for individuals with T2D almost in all the countries, the years lost due to T2D remain substantial, ranging from 2.5 to 3.1 years even in the countries with fewest years of life lost [2]. T2D has a negative impact on quality of life and increases the risk of cardiovascular disease [3, 4]. Individuals with T2D are more likely to develop coronary heart disease and the risk of myocardial infarction is over two-fold higher in individuals with T2D compared to those without diabetes [4]. Consequently, identifying modifiable risk factors for T2D should be given high priority [5]. Epidemiological and intervention studies indicate that lifestyle modifications, such as increased leisure-time physical activity, weight reduction and maintenance, and a healthy diet, can prevent or delay the onset of T2D [6].

In addition, a high level of self-reported total sitting time is associated with an increased risk of T2D, after adjustment for leisure-time physical activity [7]. A cross-sectional study found that an additional hour of daily sitting is associated with an increased risk of T2D of 22%, and an increased risk of metabolic syndrome of 39% [8]. In addition to the sitting that occurs during leisure time, many occupations today contribute to excessive total sitting time [9]. A limited number of studies have focused on context-specific sitting, such as occupational sitting, and its association with T2D risk [10]. A systematic review from 2010 found that three of four studies reported an association between occupational sitting and T2D risk [10], although the prospective studies are rare [11]. While numerous studies have examined the association between high levels of occupational physical activity and cardiovascular disease [12, 13], few have addressed the distinct impact of occupational physical activity on T2D risk [10, 11].

Interestingly, high levels of occupational physical activity do not consistently offer the same protective benefits against cardiovascular disease mortality as leisure-time physical activity [14, 15]. This phenomenon is known as the ‘Physical Activity Paradox’ [15]. Some studies have shown detrimental associations between cardiovascular mortality and high levels of occupational physical activity, especially in those with metabolic syndrome [16, 17]. Whether occupational physical activity impacts the risk of developing T2D remains unclear. Therefore, the primary aim of this study was to investigate the association between occupational physical activity and 10-year diabetes incidence in a general adult population in Northern Sweden. The secondary aim was to explore a potential moderating role of BMI on this association.

## Methods

### Study population

A cardiovascular intervention programme, the Västerbotten Intervention Program (VIP), was initiated in 1985 with the aim of reducing premature cardiovascular disease in Västerbotten County in Northern Sweden [18]. Over the years, inhabitants in Västerbotten County who turned 30 (until 1996), 40, 50, and 60 years of age have been invited to an individual risk-factor screening and a health counselling session. Blood samples are taken at the healthcare centre, with a requirement of fasting for at least 8 h beforehand. Cardiovascular biomarkers such as fasting lipids, fasting and 2-h glucose, blood pressure, and BMI are measured, and participants answer an extensive questionnaire assessing lifestyle behaviours, health aspects, and psychosocial status. Counselling with a nurse explores the participant’s lifestyle behaviours and potential for behaviour change. The use of motivational interviewing in counselling/dialogue and follow-up are fundamental parts of this intervention. Depending on the findings during the visit, necessary action, including pharmacological treatment, is taken according to current guidelines [18].

**Fig. 1:**
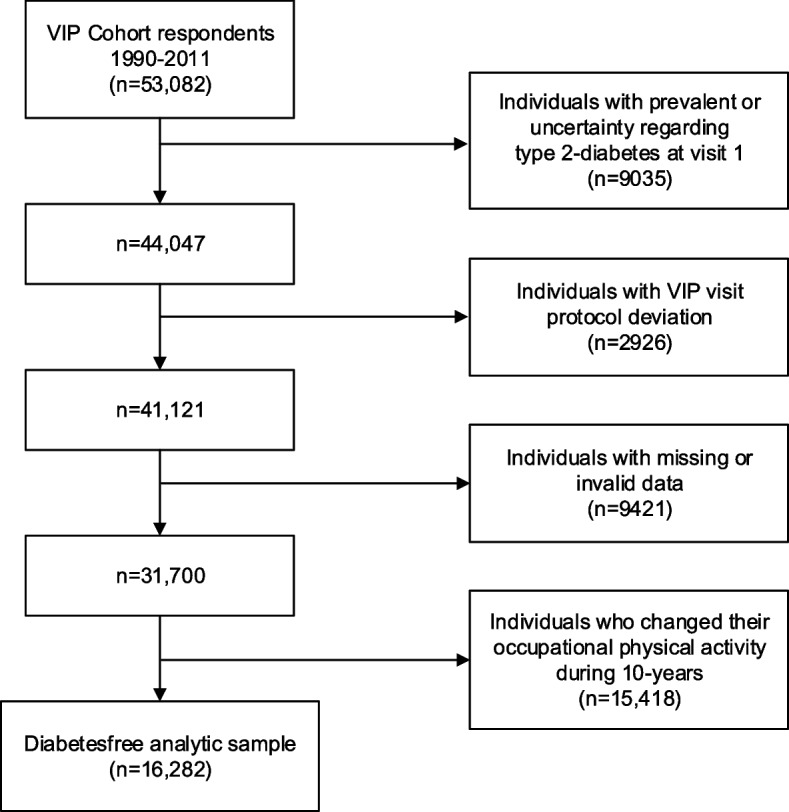
Flowchart illustrating the creation of the observational cohort

In this cohort study we included 53,082 individuals aged 28–62 years who had participated in the VIP between 1990 and 2016 and undertaken at least two VIP visits 8–12 years apart. We excluded individuals with prevalent diabetes based on self-reporting (yes/no), newly diagnosed diabetes based on first VIP visit exam (according to WHO standard), or a diabetes diagnosis in patient registers or uncertainty regarding diabetes at first VIP visit or at follow-up [19, 20]. Individuals with deviations from the VIP visit protocol were also excluded. Furthermore, we excluded individuals who had missing or invalid data for the occupational physical activity question at the first and/or second VIP visit or for other variables. Finally, we excluded individuals who had changed their occupational physical activity level in the 10 years between the first visit and the follow-up (*n* = 15,418; 48.6%), characteristics of these individuals are presented in supplementary (Table S1), leaving a total analytic sample of 16,282 individuals (Fig. 1). This study adheres to the STROBE (Strengthening the Reporting of Observational Studies in Epidemiology) guidelines for cohort studies to ensure comprehensive and transparent reporting of our research methods and findings [21].

### Incident type 2 diabetes ascertainment

The outcome of this study was incident T2D in the 10-year period between the first VIP visit and the follow-up. A high-sensitivity approach was used for the ascertainment of incident cases of T2D. The new diabetes cases were defined as 1) fasting capillary plasma glucose of ≥ 7.0 mmol/L, or a 2-h capillary plasma glucose of ≥ 12.2 mmol/L at the follow-up VIP visit or 2) a register-based diagnosis (DiabNorth) [20]. All register-based diagnoses were validated by additional blood samples, according to WHO recommendations [19].

DiabNorth is a register that links the VIP database to the patient register, the pharmacological register at the National Board of Health and Welfare, the National Diabetes Register, and medical records [19]. All of the new diabetes cases found in the DiabNorth register between the visits were included in the main analysis.

### Measurements

#### Assessment of occupational physical activity and occupational sitting

The exposure in this study was occupational physical activity, which was assessed by the following question: Which of the following options best describes your work? a) Sedentary or standing, b) Light but partly physically active, c) Light and physically active, d) Sometimes physically strenuous, or e) Physically strenuous most of the time. These five levels of occupational physical activity were modified from a four-level questionnaire originally published by Saltin and Grimby in 1968 [22]. A number of modifications to this questionnaire, which is often referred to as the Saltin-Grimby Physical Activity Level Scale (SGPALS), were used in early Scandinavian studies on leisure-time physical activity and occupational activity [23–25].

#### Covariates

Age, sex, and education level were assessed at the first VIP visit. Educational level was split into three categories: Low (mandatory schooling), Medium (secondary schooling), and High (university/college education).

Height and weight were measured while wearing light indoor clothing and without shoes, to the nearest cm and kg, respectively. Heights of < 130 cm or > 210 cm were excluded as a measurement error or data-entry error, as were weights of < 35 kg. BMI was calculated as weight (kg) divided by the square of height (m). BMIs of < 15 or > 70 were excluded as data-entry error. BMI was categorised using the clinical cut-offs for normal weight (BMI < 25 kg/m^2^) and overweight and obese (BMI ≥ 25 kg/m^2^) [26].

The Oral Glucose Tolerance Test (OGTT) was performed according to WHO standards [19]. Up to 2003, fasting and 2-h glucose were analysed using a Reflotron Bench top analyser (Roche Diagnostics) following administration of a 75 g oral glucose load on capillary plasma. Since 2004, a HemoCue® Glucose analyser (HemoCue AB, Ängelholm, Sweden) has been used*.* Prediabetes was defined as impaired fasting glucose (IFG): fasting capillary plasma-glucose 6.1–6.9 mmol/l and/or impaired glucose tolerance (IGT): 2–hour capillary plasma-glucose 8.9–12.1 [19]. Leisure-time physical activity was measured using a modified version of the short European Prospective Investigation into Cancer and Nutrition (EPIC) physical activity questionnaire (PAQ) [24], which asked the following: How often have you trained or exercised in workout clothes with the purpose of improving your fitness and/or feeling good in the last three months? a) Never, b) Every now and then – not regularly, c) 1–2 times/week, d) 2–3 times/week, or e) more than 3 times/week.

The participants also answered a food frequency questionnaire [27]. Eating two or more fruits and two or more vegetables per day was interpreted as corresponding to 500 g/day, which is the daily recommended intake internationally [28]. The following was used to define smoking status: current smoker (occasionally or daily) or non-smoker (former or never). Family history of diabetes was determined by the following question: Do any of your parents or siblings have diabetes? Ethnicity was determined by the country of birth and further classified into European or non-European origin/category.

### Statistical analyses

To examine the differences in baseline characteristics between the participants who developed diabetes during the 10-year period (cases) and those who did not (non-cases), we used a parametric test (T-test) for normally distributed variables, and a non-parametric test (Mann–Whitney U-test) for variables that were not normally distributed. Categorical variables were analysed using a Chi-squared test. If data on ‘country of birth’ or ‘family history of diabetes’ was missing for the first VIP visit, data from the follow-up visit was used.

Due to the low numbers for some of the occupational physical-activity categories, three categories instead of five were used for that variable: a) Low: ‘Sedentary or standing’ and ‘Light but partly physically active’ (*n* = 7656), b) Moderate: ‘Light and physically active’ (*n* = 3319), c) High: ‘Sometimes physically strenuous’ and ‘Physically strenuous most of the time’ (*n* = 5307), as in previous studies [16, 29].

Odds ratios (OR) and 95% confidence intervals (CI) were calculated for the association between occupational physical activity and 10-year incident diabetes, using multivariable logistic regression. ‘Moderate’ occupational physical activity (Light and physically active work) was chosen as the reference category, based on what we hypothesised would be the healthiest behaviour in accordance with previous studies [17, 29]. For the adjustments we used three models based on the assumed associations shown in Fig. S1: Model 1 was adjusted for age at first VIP visit and sex, and Model 2 was adjusted in the same way as Model 1, and for education, family history of diabetes, leisure-time physical activity, intake of fruits and vegetables, country of birth and smoking. Model 3 was adjusted for the same covariates as Model 2, for prediabetes and for BMI at the first VIP visit. We tested for interaction between BMI and occupational physical activity on T2D incidence by including an interaction term in model 3.

We used three occupational physical activity categories in our statistical analysis, which were originally derived from five categories. A sensitivity analysis was conducted with the original five occupational physical activity categories. We also explored interaction by sex and leisure-time physical activity in two secondary analyses. The percentage of missing values across the 11 variables varied between 0 (age, sex, BMI, exposure, and outcome) and 4.4% (intake of fruits and vegetables). In total, 1298 out of 17,580 records (7.4%) were incomplete and were excluded from the analysis leaving a diabetes free analytic sample of completed cases (*n* = 16,282). All analyses were carried out using IBM SPSS Statistics version 27.

## Results

After ~ 10 years (8–12 years) of follow-up, there were 624 incident diabetes cases among 16,282 individuals. The mean age at first VIP visit was 43 (SD 6.6) years. 51.5% were women, and 31% had undertaken higher education (university or college level). The mean BMI across the study population was 25.1 (3.7) kg/m^2^.

The individuals that developed T2D were older (*p* < 0.001) and had higher BMI (*p* < 0.001). They were more often men (*p* < 0.001), smokers (*p* < 0.001), had lower education (*p* < 0.001), had a family history of diabetes (*p* < 0.001), and did less physical activity during their leisure time (*p* < 0.001) (Table 1). There were no significant differences between cases and non-cases for the variables: intake of fruits and vegetables, (*p* = 0.13), country of birth (*p* = 0.31) and occupational physical activity (*p* = 0.15). Among the cases, the obesity rate was higher: 33.3%, compared to 8.7% for the non-cases (*p* < 0.001).

**Table 1:** Sociodemographic attributes, health-related and behavioural factors, and cardiometabolic risk variables of the cohort at first Västerbotten Intervention Program (VIP) visit, according to the 10-year incidence of type 2 diabetes (cases/non-cases)

**Characteristics**	**Diabetes ***(n* = *624)*	**Non-diabetes ***(n* = *15,658)*
**Age (years)**	50 ± 10	43 ± 7
**Sex (n, % male)**	370 (59.3)	7534 (48.1)
**Occupational physical activity (n, %)**		
Sedentary or standing	216 (34.6)	5204 (33.2)
Light but partly physically active	94 (15.1)	2142 (13.7)
Light and physically active	102 (16.3)	3217 (20.5)
Sometimes physically strenuous	182 (29.2)	4410 (28.2)
Physically strenuous most of the time	30 (4.8)	685 (4.4)
**Occupational physical activity levels (n, %)**		
Low	310 (49.7)	7346 (46.9)
Moderate	102 (16.3)	3217 (20.5)
High	212 (34.0)	5095 (32.5)
**Educational level (n, %)**		
Low (≤ 9 years)	303 (48.6)	5168 (33.0)
Medium (10–12 years)	199 (31.9)	5564 (35.5)
High (≥ 13 years)	122 (19.6)	4926 (31.5)
**Family history of diabetes** **(n, %)**		
Yes	206 (33.0)	2645 (16.9)
No	418 (67.0)	13 013 (83.1)
**Weight (kg)**	85.0 ± 16.5	74.4 ± 13.8
**BMI (kg/m**^**2**^**) Visit 1**	28.5 ± 4.6	24.5 ± 4.4
Normal weight (≤ 24.99 kg/m^2^)	153 (24.5)	8669 (55.4)
Overweight (25–29.9 kg/m^2^)	263 (42.1)	5626 (35.9)
Obese (≥ 30 kg/m^2^)	208 (33.3)	1363 (8.7)
**Prediabetes (n, %)**	277 (44.4)	1552 (9.9)
**Smoking** **(n, %)**		
Never/Former smoker	456 (73.1)	12 471 (79.6)
Current (smoker)	168 (26.9)	3187 (20.4)
**Leisure-time physical activity (n, %)**		
Never	307 (49.2)	5921 (37.8)
Every now and then – not regularly	162 (26.0)	4006 (25.6)
1–2 times/week	94 (15.1)	3177 (20.3)
2–3 times/week	46 (7.4)	1851 (11.8)
More than 3 times/week	15 (2.4)	703 (4.5)
**Fruits and vegetables (n, %)**		
< 2 portions/day	588 (94.2)	14 504 (92.6)
≥ 2 portions/day	36 (5.8)	1154 (7.4)
**Country of birth** **(n, %)**		
European	618 (99.0)	15 559 (99.4)
Non-European	6 (1.0)	99 (0.6)
**Follow-up time (years)**	9.93 (0.31)	9.93 (0.32)
Continuous parametric results as mean ± SD, number (percentage) and continuous nonparametric results as median (interquartile range). Occupational physical activity levels: Low: ‘Sedentary or standing’ and ‘Light but partly physically active’; Moderate: ‘Light and physically active’ and High: ‘Sometimes physically strenuous’ and ‘Physically strenuous most of the time’		

Across the sample, a low level of occupational physical activity, compared with a moderate level of occupational physical activity, was associated with an increased risk of incident T2D after adjustment for covariates in Model 2 (OR 1.30, 95% CI 1.03–1.64), but this association was attenuated and no longer significant after adjustment for BMI and prediabetes in Model 3 (OR 1.24, 95% CI 0.98–1.58).

In Model 3, the interaction between BMI categories and occupational physical activity was significant (*p* = 0.027); therefore, the data was reported in two BMI categories: < 25 kg/m^2^, and ≥ 25 kg/m^2^. In individuals with BMI of ≥ 25 kg/m^2^, a low level of occupational physical activity, compared with a moderate level of occupational physical activity, was associated with an increased risk of incident T2D (Model 3, OR 1.46, 95% CI 1.09–1.96); this was not the case for those with a BMI of < 25 kg/m^2^ (OR 0.80, 95% CI 0.52–1.23) (Table 2). There was no significant association between a high level of occupational physical activity and risk of T2D.

**Table 2:** Association between type 2 diabetes risk and long-term occupational physical activity. *n* = 16,282, 624 diabetes cases

**Occupational physical** **activity level**		**Model 1**^**a**^		**Model 2**^**b**^		**Model 3**^**c**^	
		**OR**	**95% CI**	**OR**	**95% CI**	**OR**	**95% CI**
***BMI* < *25 ***	***DiabetesCases***						
** Moderate (***n* **= 1960)**	*n* = 36	ref		ref		ref	
** Low (***n* **= 4058)**	*n* = 58	0.77	0.51 to 1.18	0.77	0.50 to 1.18	0.80	0.52 to 1.23
** High (***n* **= 2804)**	*n* = 59	1.18	0.78 to 1.80	0.88	0.57 to 1.36	0.88	0.56 to 1.36
***BMI* ≥ *25***							
** Moderate (***n* **= 1359)**	*n* = 66	ref		ref		ref	
** Low (***n* **= 3598)**	*n* = 252	1.49	1.13 to 1.97	1.47	1.11 to 1.95	1.46	1.09 to 1.96
** High (***n* **= 2503)**	*n* = 153	1.36	1.10 to 1.83	1.16	0.86 to 1.58	1.12	0.82 to 1.54

^a^Model 1 was adjusted for age and sex at Visit 1

^b^Model 2 was adjusted for age, sex, education, family history of diabetes, leisure-time physical activity, intake of fruits and vegetables, birth country and smoking at Visit 1

^c^Model 3 was adjusted for the same covariates as Model 2, prediabetes and BMI at Visit 1

No significant interaction was observed between sex and occupational physical activity (*p* = 0.58) or between leisure-time physical activity and occupational physical activity (*p* = 0.73) (Table S2-S3).

The results were robust in sensitivity analyses of occupational physical activity in five categories, with significant associations between both ‘Sedentary or standing’ and ‘Light but partly physically active’, as compared to ‘Light and physically active’ (reference category), and risk of incident T2D. Similarly, there was no significant association between the other levels of occupational physical activity and risk of T2D (Supplementary Table S4).

## Discussion

This cohort study found that overweight and obese individuals with low levels of occupational physical activity, which was measured twice in a 10-year period, had an increased risk of developing T2D. The association seemed to be moderated by BMI, and occupational physical activity was not associated with T2D risk in individuals with normal weight. The secondary analysis indicated a significant association between low level of occupational physical activity and T2D only among men with overweight and obesity. There was no significant sex interaction, however, and therefore the sex-specific findings should be interpreted with caution. This study contributes with evidence that consistently low occupational physical activity may contribute to T2D risk. However, the association between low levels of occupational physical activity and T2D risk was only observed in overweight and obese individuals. Previous research on the VIP cohort has indicated a reduced risk of T2D among individuals who maintained their weight over a 10-year period [30]. Our study provides further support for the idea that maintaining a normal weight offers protection against the long-term harms of sedentary work in relation to developing T2D. Our results are in line with those of the cross-sectional EPIC-Norfolk study [31] and the prospective Nurses’ Health Study [32], both of which found that sedentary work was associated with an increased risk of T2D. In the prospective Whitehall cohort, an association between occupational sitting and T2D was found, but this association was attenuated after adjustment for baseline BMI [33]. In contrast, a prospective study of a Japanese cohort [34] did not find a significant risk of T2D in participants with low occupational physical activity, but the analysis may have been limited by a single assessment of occupational physical activity and short follow-up time (6 years).

Obesity is known to be the most important risk factor for T2D, and the association between BMI and incident diabetes has been found to be approximately linear [35, 36].

A WHO report published in 2022 noted that obesity rates in Europe have continued to rise, with 59% of adults overweight or obese at the time of publication [37]. An analysis of occupational physical activity and energy expenditure trends in the USA over the last 50 years have shown a clear shift away from high and moderate levels of occupational physical activity, and towards lighter and sedentary behaviour at work [9]. Furthermore, a decrease in occupation-related energy expenditure of more than 100 cal per person per day was identified, which is suggested to have contributed to weight gain in that population [9].

Experimental research has provided insights regarding the benefits of interrupting prolonged sitting with short breaks, showing a lowering of the postprandial insulin response in overweight and obese individuals [38]. A similar response was not found in adults with normal weight [39]. These findings suggest that interrupting prolonged sitting may be more beneficial for the glucose metabolisms of overweight and obese individuals.

The Physical Activity Paradox suggests that while leisure-time activity improves cardiovascular health, very high levels of physical activity at work can be detrimental [13]. One of the hypotheses to explain the Paradox is that occupational physical activity is often of too low intensity to improve cardiovascular fitness and have a positive effect on health [16]. Further findings that contrast the Physical Activity Paradox have been reported in a systematic review and meta-analysis, showing a 15% lower T2D risk for high occupational physical activity as compared to low levels [11]. In the current study, we did not find an association between a high level of occupational physical activity and a reduced risk of T2D compared to moderate levels of occupational physical activity. Therefore, the lack of protective effects of high occupational physical activity in lowering diabetes risk supports the Physical Activity Paradox.

### Strengths and limitations

This was a prospective cohort study using data from a large, general population of both men and women, with a long follow-up period. The study population includes participants from diverse occupational backgrounds minimizing the risk of selection bias. The longitudinal design enabled us to assess the exposure and outcome, as well as the potential moderating role of BMI, over time. Another strength was that a high-sensitivity approach was used to identify the new cases of T2D, with the intention of identifying all the incident cases during the follow-up period. Moreover, we had access to comprehensive data for adjustment for a wide variety of covariates. This may lead to over adjustment in the fully adjusted model (Model 3) as some of the confounders may also act as mediators such as prediabetes. A sensitivity analysis confirmed the robustness of our findings.

It is important to note that participants in this study were involved in an individual intervention program (VIP) targeting multiple risk factors during their initial visit [18]. This may have resulted in changes to their lifestyle habits over the 10-year period, potentially introducing measurement bias for covariates. However, it is not expected to affect the exposure variable, occupational physical activity.

We used self-reported physical activity, which could lead to misclassification. To ensure that the exposure to occupational physical activity was consistent over a long period of time and to reduce the risk of misclassification, only the participants who reported the same occupational physical activity level at baseline and the 10-year follow-up were included in the analysis.

However, as the individuals who had changed their occupational physical activity level in the 10 years between the first visit and the follow-up were excluded, this may limit the generalizability of our findings. Furthermore, we cannot rule out the possibility of residual confounding as information about shift work and job strain which has been shown to be associated with T2D risk, was lacking [40, 41]. We adjusted for daily intake of fruits and vegetables, but there may be other dietary factors that are of relevance to T2D risk that we did not adjust for.

### Clinical interpretation

Increased leisure-time physical activity is a cornerstone in the prevention of T2D. In addition, by recognizing low occupational physical activity as a significant risk factor for T2D in overweight and obese individuals, healthcare providers can implement more personalized prevention strategies. For example, increased occupational physical activity could be encouraged when it is practically possible, particularly in persons with sedentary work and overweight or obesity. On the population level, public-health efforts may benefit from focusing on work environments where workers spend a lot of time sitting, to promote less sitting and standing, and instead encouraging more light physical activity. Many employers are already implementing interventions in workplaces to decrease sitting time among their employees, which may present possibilities for research such as natural experiments [42].

## Conclusion

Overweight and obese individuals who reported a consistently low level of occupational physical activity had an increased risk of T2D compared to those that reported moderate level of occupational physical activity. Consequently, implementing strategies to reduce occupational sitting among overweight and obese individuals – such as incorporating light physical activities during the workday – could be a suitable target for public-health interventions.

## Supplementary Information

Supplementary Material 1.

## Data Availability

Access to individual-level data can be provided for research purposes but is restricted by laws regarding the privacy of research participants and therefore not made publicly available. Requests for data can be sent to the Section of Biobank and Registry Support at Umeå University (info.brs@ umu.se).

## Abbreviations

BMI: Body Mass Index
CI: Confidence interval
EPIC: European Prospective Investigation into Cancer and Nutrition
OGTT: Oral Glucose Tolerance Test
OR: Odds ratio
PAQ: Physical activity questionnaire
SD: Standard deviation
SGPALS: Saltin-Grimby Physical Activity Level Scale
TD2: Type 2 diabetes
VIP: Västerbotten Intervention Program
WHO: World Health Organization
