# Supplementary material for: Low occupational physical activity is associated with incident type 2 diabetes in overweight and obese individuals: a population-based cohort study
Source: BMC Public Health. 2025 Apr 14;25:1389. doi: 10.1186/s12889-025-22534-5 (PMC11995627; doi:10.1186/s12889-025-22534-5)
Supplement: Supplementary file 1 — Supplementary Material 1. [file 12889_2025_22534_MOESM1_ESM.docx]

**Supplementary**

**Low occupational physical activity is associated with incident type 2 diabetes in overweight and obese individuals: A population-based cohort study**

**Authors**: Maria Brännholm Syrjälä^1^, Melony Fortuin-De Smidt^1^, Frida Bergman^1^, Maria Nordendahl^1^, Julia Otten^1^, Rebecka Renklint^1^, Olov Rolandsson^1^, Viktoria Wahlström^1^, Patrik Wennberg^1^

**Institutions:** ^1^ Department of Public Health and Clinical Medicine, Umeå University, Umeå, Sweden

**Table S1**. Sociodemographic attributes, health-related and behavioural factors, and cardiometabolic risk variables of the cohort at first Västerbotten Intervention Program (VIP) visit, according to the 10-year incidence of type 2 diabetes (cases/non-cases). Characteristics of those with stable occupational physical activity level (included in main analysis, n= 16,282) and those who changed their occupational physical activity during the 10-year period (excluded, n=15,418).

|  | **Included**  **(n=16,282)** | | **Excluded**  **(n=15,418)** | |
| --- | --- | --- | --- | --- |
| **Characteristics** | **Diabetes**  *(n=624)* | **Non-diabetes**  *(n=15,658)* | **Diabetes**  *(n=600)* | **Non-diabetes**  *(n=14,818)* |
| **Age (years)** | 50±10 | 43±7 | 46±6 | 43±7 |
| **Sex (n, % male)** | 370 (59.3) | 7534 (48.1) | 355 (59.2) | 7039 (47.5) |
| **Occupational physical activity (n, %)**        Sedentary or standing        Light but partly physically active        Light and physically active        Sometimes physically strenuous        Physically strenuous most of the time | 216 (34.6)  94 (15.1)  102 (16.3)  182 (29.2)  30 (4.8) | 5204 (33.2)  2142 (13.7)  3217 (20.5)  4410 (28.2)  685 (4.4) | 132 (22.0)  146 (24.3)  132 (22.0)  145 (24.2)  45 (7.5) | 2837 (19.1)  3456 (23.3)  3698 (25.0)  3715 (25.1)  1112 (7.5) |
| **Occupational physical activity levels (n, %)**  Low  Moderate  High | 310 (49.7)  102 (16.3)  212 (34.0) | 7346 (46.9)  3217 (20.5)  5095 (32.5) | 278 (46.3)  132 (22.0)  190 (31.7) | 6293 (42.4)  3698 (25.0)  4827 (32.6) |
| **Educational level (n, %)**        Low (≤9 years)        Medium (10–12 years)        High (≥13 years) | 303 (48.6)  199 (31.9)  122 (19.6) | 5168 (33.0)  5564 (35.5)  4926 (31.5) | 283 (47.2)  206 (34.3)  111 (18.5) | 5173 (34.9)  5633 (38.0)  4012 (27.1) |
| **Family history of diabetes** **(n, %)**        Yes        No | 206 (33.0)  418 (67.0) | 2645 (16.9)  13 013 (83.1) | 198 (33.0)  402 (67.0) | 2568 (17.3)  12 250 (82.7) |
| **Weight (kg)** | 85.0±16.5 | 74.4±13.8 | 85.1±16.3 | 74.4±13.6 |
| **BMI (kg/m^2^) Visit 1**        Normal weight (*≤*24.99 kg/m^2^)        Overweight (25–29.9 kg/m^2^)        Obese (≥30 kg/m^2^) | 28.5±4.6  153 (24.5)  263 (42.1)  208 (33.3) | 24.5±4.4  8669 (55.4)  5626 (35.9)  1363 (8.7) | 28.5±4.6  132 (22.0)  285 (47.5)  132 (22.0) | 24.6±4.5  8002 (54.0)  5446 (36.8)  1370 (9.2) |
| **Prediabetes (n, %)** | 277 (44.4) | 1552 (9.9) | 267 (44.5) | 1460 (9.9) |
| **Smoking** **(n, %)**        Never/Former smoker        Current (smoker) | 456 (73.1)  168 (26.9) | 12 471 (79.6)  3187 (20.4) | 437 (72.8)  163 (27.2) | 11 619 (78.4)  3199 (21.6) |
| **Leisure-time physical activity (n, %)**        Never  Every now and then – not regularly  1–2 times/week  2–3 times/week  More than 3 times/week | 307 (49.2)  162 (26.0)  94 (15.1)  46 (7.4)  15 (2.4) | 5921 (37.8)  4006 (25.6)  3177 (20.3)  1851 (11.8)  703 (4.5) | 291 (48.5)  166 (27.7)  82 (13.7)  35 (5.8)  26 (3.9) | 5945 (40.1)  3794 (25.6)  2807 (18.9)  1595 (10.8)  677 (4.6) |
| **Fruits and vegetables (n, %)**        <2 portions/day  *≥*2 portions/day | 588 (94.2)  36 (5.8) | 14 504 (92.6)  1154 (7.4) | 560 (93.3)  40 (6.7) | 13 756 (92.8)  1062 (7.2) |
| **Country of birth** **(n, %)**      European      Non-European | 618 (99.0)  6 (1.0) | 15 559 (99.4)  99 (0.6) | 585 (97.5)  15 (2.5) | 14 629 (98.7)  189 (1.3) |
| **Follow-up time (years)** | 9.93 (0.31) | 9.93 (0.32) | 9.93 (0.31) | 9.95 (0.28) |
| *Continuous parametric results as mean ±SD, number (percentage) and continuous nonparametric results as median (interquartile range).*  *Occupational physical activity levels:* *Low: ‘Sedentary or standing’ and ‘Light but partly physically active’; Moderate: ‘Light and physically active’ and High: ‘Sometimes physically strenuous’ and ‘Physically strenuous most of the time’.* | | | | |

**A**


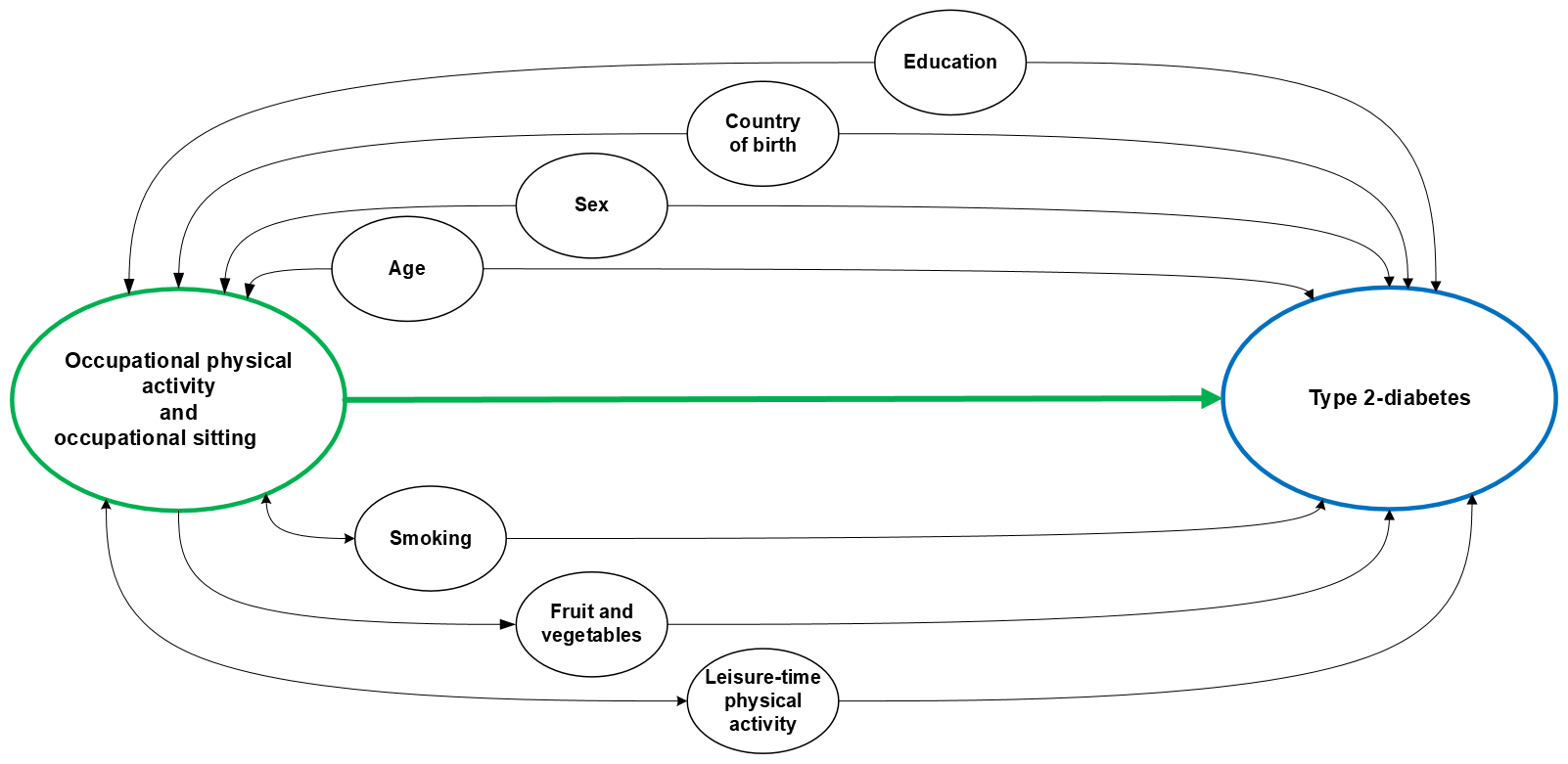


**B**


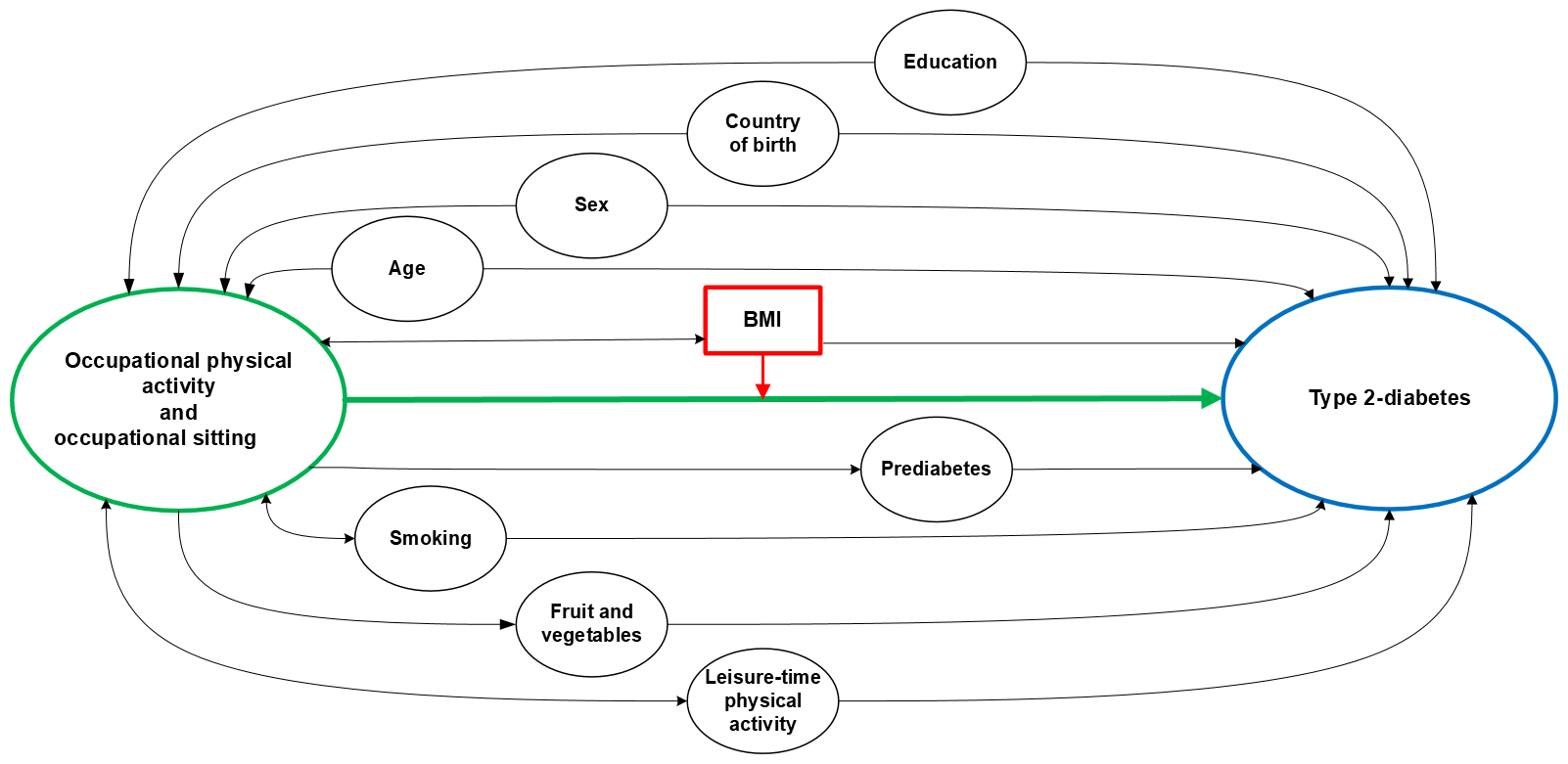


**Figure S1.** Directed acyclic graphs (DAGs) describing the potential causal pathways of occupational physical activity and occupational sitting on T2D incidence. Circles: Green= exposure, Blue = outcome, Black = ancestor of exposure and outcome. Arrows: Green = causal path. Square: Red = moderator.

A) DAG with potential confounders adjusted in Model 2: age, sex, education, family history of diabetes, leisure-time physical activity, intake of fruits and vegetables, country of birth and smoking. Some confounders may have a stronger effect on the outcome (T2D), but only a weak effect on the exposure (e.g. intake of fruit and vegetables) and could also be considered as mediators. B) DAG describing Model 3 with potential confounders as in Model 2, plus BMI and Prediabetes. BMI is assumed to be both a moderator and confounder but could also be a mediator. Prediabetes is also a potential mediator, which may lead to over-adjustment in the Model 3.

**Table S2.** Association between type 2 diabetes risk and occupational physical activity between first VIP visit and 10-year follow up.

Stratification by sex and BMI category.

| **Sex** | **Occupational physical**  **activity level** | **Model 1^a^** | | **Model 2^b^** | | **Model 3^c^** | |
| --- | --- | --- | --- | --- | --- | --- | --- |
|  |  | **OR** | **95% CI** | **OR** | **95% CI** | **OR** | **95% CI** |
| ***Female*** | ***BMI <25*** |  |  |  |  |  |  |
|  | Moderate (n=1319) | ref. |  | ref. |  | ref. |  |
|  | Low (n=2391) | 0.87 | 0.49 to 1.53 | 0.73 | 0.41 to 1.30 | 0.76 | 0.43 to 1.36 |
|  | High (n=1565) | 1.14 | 0.63 to 2.05 | 0.79 | 0.43 to 1.45 | 0.78 | 0.42 to 1.45 |
| ***Female*** | ***BMI ≥25*** |  |  |  |  |  |  |
|  | Moderate (n=671) | ref. |  | ref. |  | ref. |  |
|  | Low (n=1383) | 1.50 | 0.98 to 2.32 | 1.36 | 0.88 to 2.11 | 1.24 | 0.79 to 1.96 |
|  | High (n=1049) | 1.37 | 0.87 to 2.16 | 0.95 | 0.62 to 1.59 | 0.85 | 0.52 to 1.39 |
| ***Male*** | ***BMI <25*** |  |  |  |  |  |  |
|  | Moderate (n=641) | ref. |  | ref. |  | ref. |  |
|  | Low (n=1667) | 0.68 | 0.36 to 1.27 | 0.79 | 0.41 to 1.50 | 0.80 | 0.41 to 1.54 |
|  | High (n=1239) | 1.21 | 0.66 to 2.23 | 0.98 | 0.53 to 1.82 | 0.96 | 0.51 to 1.80 |
| ***Male*** | ***BMI ≥25*** |  |  |  |  |  |  |
|  | Moderate (n=688) | ref. |  | ref. |  | ref. |  |
|  | Low (n=2215) | 1.49 | 1.03 to 2.16 | 1.50 | 1.03 to 2.19 | 1.56 | 1.05 to 2.30 |
|  | High (n=1454) | 1.37 | 0.92 to 2.03 | 1.25 | 0.84 to 1.87 | 1.30 | 0.85 to 1.99 |

a Model 1 was adjusted for age at Visit 1.

b Model 2 was adjusted for age, education, family history of diabetes, leisure-time physical activity, intake of fruits and vegetables, birth country and smoking at Visit 1.

c Model 3 was adjusted for the same covariates as Model 2, prediabetes and BMI at Visit 1.

**Table S3.** Association between type 2 diabetes risk and occupational physical activity between first VIP visit and 10-year follow up.

Stratification by leisure-time physical activity level and BMI category.

| **Leisure-time physical activity level** | **Occupational physical**  **activity level** | **Model 1^a^** | | **Model 2^b^** | | **Model 3^c^** | |
| --- | --- | --- | --- | --- | --- | --- | --- |
|  |  | **OR** | **95% CI** | **OR** | **95% CI** | **OR** | **95% CI** |
| ***≤ 1–2 times/week*** | ***BMI <25*** |  |  |  |  |  |  |
|  | Moderate (n=1621) | ref. |  | ref. |  | ref. |  |
|  | Low (n=3262) | 0.84 | 0.53 to 1.34 | 0.81 | 0.51 to 1.30 | 0.86 | 0.53 to 1.38 |
|  | High (n=2352) | 1.22 | 0.77 to 1.95 | 0.94 | 0.58 to 1.51 | 0.92 | 0.57 to 1.49 |
| ***≤ 1–2 times/week*** | ***BMI ≥25*** |  |  |  |  |  |  |
|  | Moderate (n=1144) | ref. |  | ref. |  | ref. |  |
|  | Low (n=3076) | 1.41 | 1.06 to 1.89 | 1.40 | 1.05 to 1.88 | 1.24 | 0.79 to 1.96 |
|  | High (n=2212) | 1.27 | 0.93 to 1.73 | 1.12 | 0.82 to 1.53 | 0.85 | 0.52 to 1.39 |
| ***≥ 2–3 times/week*** | ***BMI <25*** |  |  |  |  |  |  |
|  | Moderate (n=339) | ref. |  | ref. |  | ref. |  |
|  | Low (n=796) | 0.46 | 0.17 to 1.29 | 0.50 | 0.18 to 1.38 | 0.55 | 0.19 to 1.58 |
|  | High (n=452) | 1.02 | 0.37 to 2.80 | 0.70 | 0.25 to 2.01 | 0.81 | 0.27 to 2.38 |
| ***≥ 2–3 times/week*** | ***BMI ≥25*** |  |  |  |  |  |  |
|  | Moderate (n=215) | ref. |  | ref. |  | ref. |  |
|  | Low (n=522) | 2.44 | 0.83 to 7.19 | 2.32 | 0.77 to 7.05 | 2.80 | 0.89 to 8.80 |
|  | High (n=291) | 2.16 | 0.66 to 7.03 | 1.99 | 0.59 to 6.74 | 2.20 | 0.62 to 7.83 |

a Model 1 was adjusted for age and sex at Visit 1.

b Model 2 was adjusted for age, sex, education, family history of diabetes, intake of fruits and vegetables, birth country and smoking at Visit 1.

c Model 3 was adjusted for the same covariates as Model 2, prediabetes and BMI at Visit 1.

**Table S4.** Association between type 2 diabetes risk and occupational physical activity. n=16,282, n=624 diabetes cases. Sensitivity analyses of occupational physical activity in five categories.

|  | **Model 1^a^** | | **Model 2^b^** | | **Model 3^c^** | |
| --- | --- | --- | --- | --- | --- | --- |
| **Occupational physical activity category** | **OR** | **95% CI** | **OR** | **95% CI** | **OR** | **95% CI** |
| **BMI <25** |  |  |  |  |  |  |
| **Light and physically active (n=1960)** | ref. |  | ref. |  | ref. |  |
| **Sedentary or standing** **(n=2891)** | 0.85 | 0.55-1.33 | 0.83 | 0.53-1.30 | 0.86 | 0.55-1.35 |
| **Light but partly physically active** **(n=1167)** | 0.58 | 0.30-1.09 | 0.62 | 0.32-1.17 | 0.65 | 0.34-1.25 |
| **Sometimes physically straining (n=2456)** | 1.17 | 0.76–1.81 | 0.88 | 0.57-1.38 | 0.88 | 0.56-1.38 |
| **Physically straining most of the time (n=348)** | 1.28 | 0.59-2.78 | 0.87 | 0.39-1.91 | 0.86 | 0.39-1.92 |
| **BMI ≥25** |  | |  | |  | |
| **Light and physically active** **(n=1359)** | ref. |  | ref. |  | ref. |  |
| **Sedentary or standing (n=2529)** | 1.47 | 1.09-1.97 | 1.45 | 1.08-1.95 | 1.44 | 1.06-1.96 |
| **Light but partly physically active (n=1069)** | 1.54 | 1.10-2.16 | 1.51 | 1.07-2.12 | 1.52 | 1.07-2.17 |
| **Sometimes physically straining (n=2136)** | 1.37 | 1.01-1.86 | 1.17 | 0.86-1.60 | 1.13 | 0.82-1.57 |
| **Physically straining most of the time** **(n=367)** | 1.31 | 0.80-2.17 | 1.10 | 0.67-1.83 | 1.08 | 0.64-1.82 |

a Model 1 was adjusted for age and sex at Visit 1.

b Model 2 was adjusted for age, sex, education, family history of diabetes, leisure-time physical activity, intake of fruits and vegetables, birth country and smoking at

Visit 1.

c Model 3 was adjusted for the same covariates as Model 2, prediabetes and BMI at Visit 1.
